# Use of Diperoxochloric Acid as a Novel Therapy for the Treatment of Infected Traumatic Foot Ulcers: A Case Series

**DOI:** 10.7759/cureus.90895

**Published:** 2025-08-24

**Authors:** Haritha Shahnaz Masthan, Samir Ahmad, Bharathi Raja K, N Guru Prasad, Santhaseelan R G

**Affiliations:** 1 General Surgery, Sree Balaji Medical College, Chennai, IND; 2 General Surgery, Sree Balaji Medical College and Hospital, Chennai, IND

**Keywords:** antimicrobial therapy, debridement, diperoxochloric acid, infection control, topical antiseptic, traumatic foot ulcer, wound healing

## Abstract

Infected traumatic foot ulcers present a significant therapeutic challenge, particularly in patients with delayed presentation, suboptimal hygiene, or comorbidities like diabetes and peripheral vascular disease. Conventional management includes wound debridement, systemic antibiotics, and advanced dressings, often with variable success. Diperoxochloric acid (DPA), a potent oxidizing agent with broad-spectrum antimicrobial properties, has recently gained attention for its potential in promoting wound healing while simultaneously reducing the microbial load. In this case series, we present four patients with moderate to severe infected foot ulcers following trauma, all of whom showed minimal improvement after at least 14 days of standard wound care and systemic antibiotic therapy. Upon introduction of topical DPA as an adjunctive treatment along with serial wound debridement, all cases demonstrated significant clinical improvement, with a rapid reduction in wound size (average decrease of 60% within three weeks), resolution of infection, and progressive granulation leading to complete healing in an average of 6.5 weeks. Compared to traditional antiseptics, DPA offers several advantages, including reduced cytotoxicity, sustained antimicrobial activity, and enhanced wound bed preparation, making it a compelling adjunct in chronic and infected wound care. This series highlights the potential of DPA as a cost-effective, efficient, and well-tolerated adjunct in the multidisciplinary management of infected traumatic foot ulcers. Future prospective studies with larger cohorts are warranted to further elucidate optimal protocols, long-term outcomes, and cost-effectiveness.

## Introduction

Traumatic foot ulcers represent a significant clinical challenge due to their propensity for contamination, delayed healing, and progression to deep soft‑tissue infection. These wounds often arise from minor injuries, such as stepping on sharp objects or falls, and can rapidly become colonized by pathogenic bacteria if not managed promptly. Oxidative antiseptics have emerged as valuable adjuncts in wound care, offering broad‑spectrum antimicrobial activity while preserving host tissue integrity; in particular, diperoxochloric acid (DPA), a peracid compound with potent bactericidal and fungicidal properties [1]. It is seen affecting an estimated X per 100,000 individuals annually and often leading to prolonged disability or hospitalization.

The modern approach to wound management is encapsulated by the TIME framework, which highlights four critical components: Tissue debridement, Infection control, Moisture balance, and Edge advancement [2]. Selective surgical or bedside debridement removes nonviable tissue, while topical agents, such as DPA, reduce microbial bioburden and disrupt biofilms. Maintaining an optimal moisture environment further supports granulation and epithelial migration, and ensuring healthy wound edges promotes rapid closure [2].

The TIME framework, widely adopted across both acute and chronic wound care, provides a structured approach to non‑diabetic traumatic lesions [3]. Comparative analyses of commonly used antiseptics have highlighted differences in cytotoxicity and spectrum of action; for example, povidone‑iodine and hydrogen peroxide can impair fibroblast function at higher concentrations, whereas peracid compounds like DPA achieve microbial kill with minimal host‑cell damage [4]. Additionally, the pH of the wound environment modulates the activity of antiseptic agents, with mildly acidic conditions favoring healing and enhancing the antimicrobial efficacy of oxidizing agents such as DPA [5,6]. Although much of the research has centered on diabetic foot ulcers, the underlying wound-healing principles and antiseptic strategies are equally relevant to non-diabetic traumatic lesions.

Despite these insights, there remains a lack of case series evaluating DPA in the management of traumatic foot ulcers. Here, we describe four cases of infected traumatic foot ulcers treated with serial debridement and topical DPA, with demonstrated acceleration of healing and effective infection control. Such a case series provides valuable real-world insights into treatment responses, safety, and feasibility.

## Case presentation

Case 1

**Figure 1:**
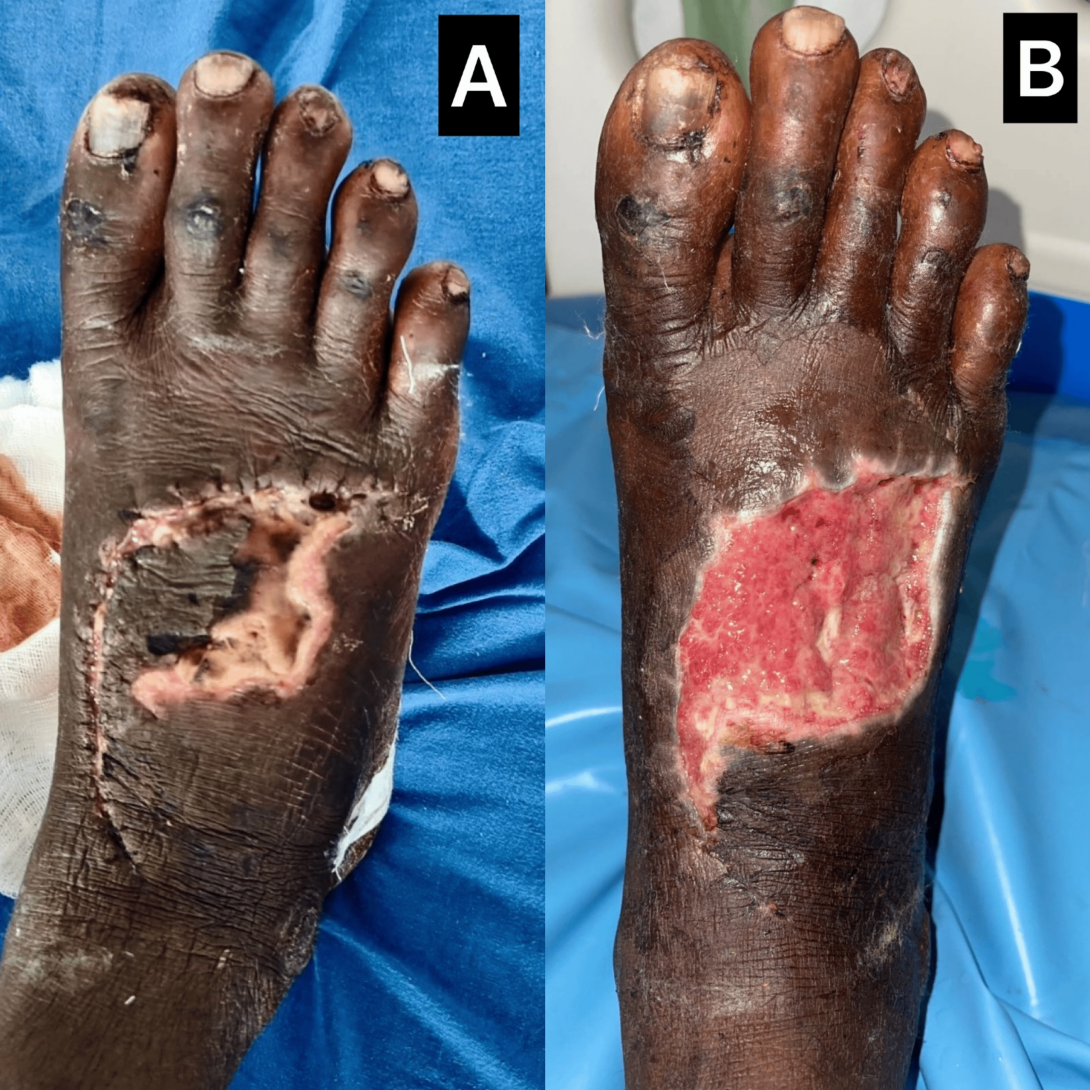
Progression of dorsal foot ulcer healing with DPA therapy 1A: Infected traumatic ulcer with slough, exposed tendons, and perilesional necrosis on the dorsum of the foot at presentation; 1B: Healed wound after serial debridement and topical diperoxochloric acid application, showing healthy granulation and contraction DPA: diperoxochloric acid

A 60-year-old male farmer presented with a traumatic ulcer over the dorsum of the left foot after stepping on a sharp metallic object. The patient delayed seeking medical care and arrived five days post-injury with increasing pain, swelling, and foul-smelling discharge. Examination revealed a 6 × 4 cm ulcer with an irregular base covered in slough, exposed extensor tendons, and perilesional necrotic skin changes as shown in Figure 1. Peripheral pulses were intact. Wound swab culture showed *Staphylococcus aureus* sensitive to ceftriaxone, which was initiated. Ceftriaxone was continued for a total duration of 10 days. The patient underwent surgical debridement under regional anesthesia, followed by daily dressings using gauze soaked in diperoxochloric acid (DPA). Two additional debridements were performed on days 4 and 9. By the third week, the wound exhibited healthy granulation tissue, reduced size, and no discharge. Complete healing was achieved by the fifth week without complications.

Case 2

**Figure 2:**
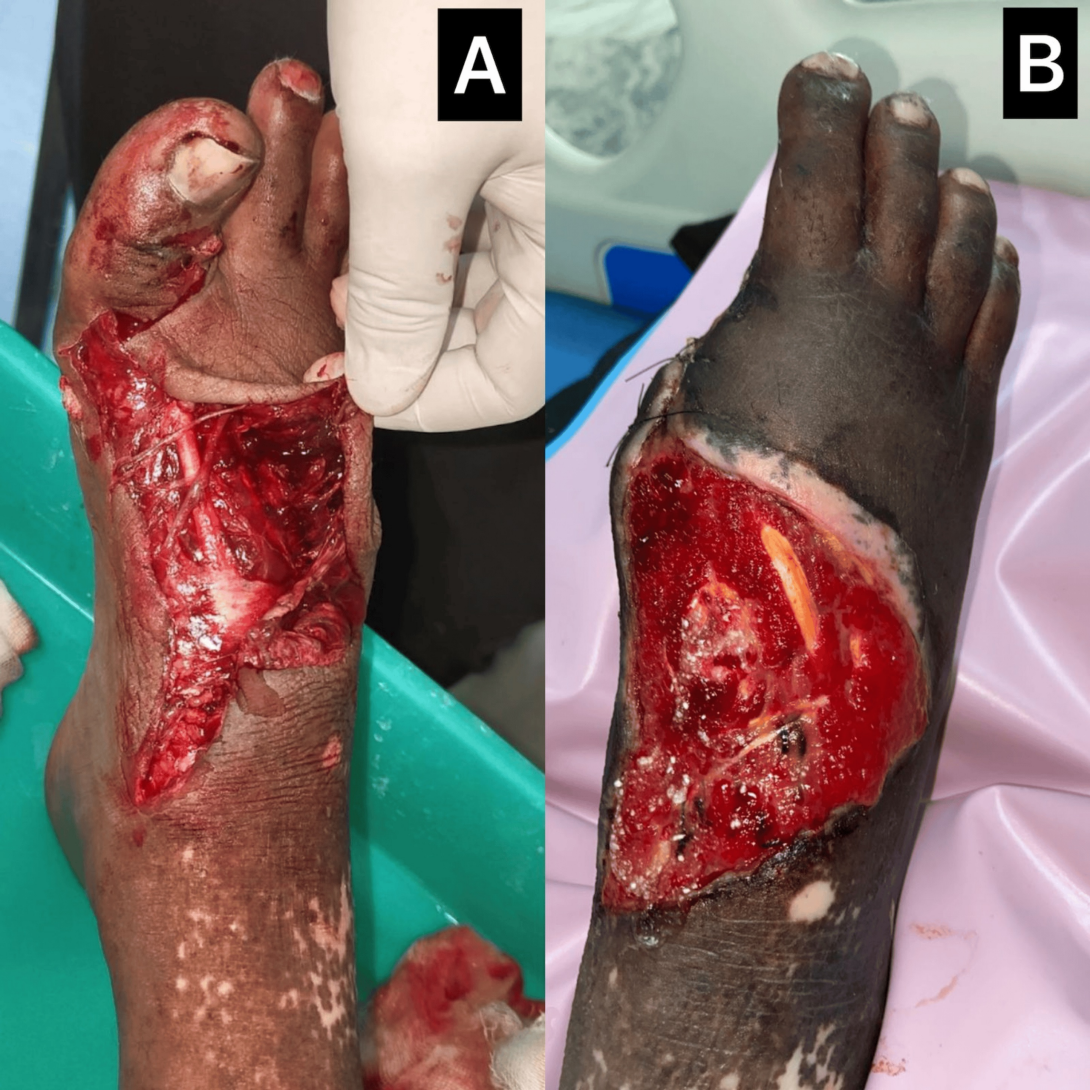
Deep dorsal laceration healing following DPA application 2A: Large, gaping wound with exposed extensor tendons and macerated edges after initial trauma and debridement; 2B: Significant granulation tissue and wound contraction seen after four weeks of DPA therapy and wound care DPA: diperoxochloric acid

A 47-year-old male presented with a deep laceration over the dorsum of the right foot following a road traffic accident. On examination, the wound measured 7 × 5 cm with exposed tendons, irregular margins, and moderate purulent discharge as shown in Figure 2. The patient was hemodynamically stable and had no comorbidities. Surgical debridement was performed on the day of admission. Weight-bearing on the affected limb was restricted during the initial two weeks to prevent mechanical disruption of the wound. Wound cultures later grew *Klebsiella pneumoniae* sensitive to amikacin, which was added to intravenous antibiotics. Topical wound management with DPA dressings was initiated immediately and continued daily. Granulation tissue appeared within the first week. Two minor debridements were performed during follow-up to remove residual slough. By the fourth week, the wound had contracted significantly with near-complete epithelialization. The patient resumed weight-bearing without complications.

Case 3

**Figure 3:**
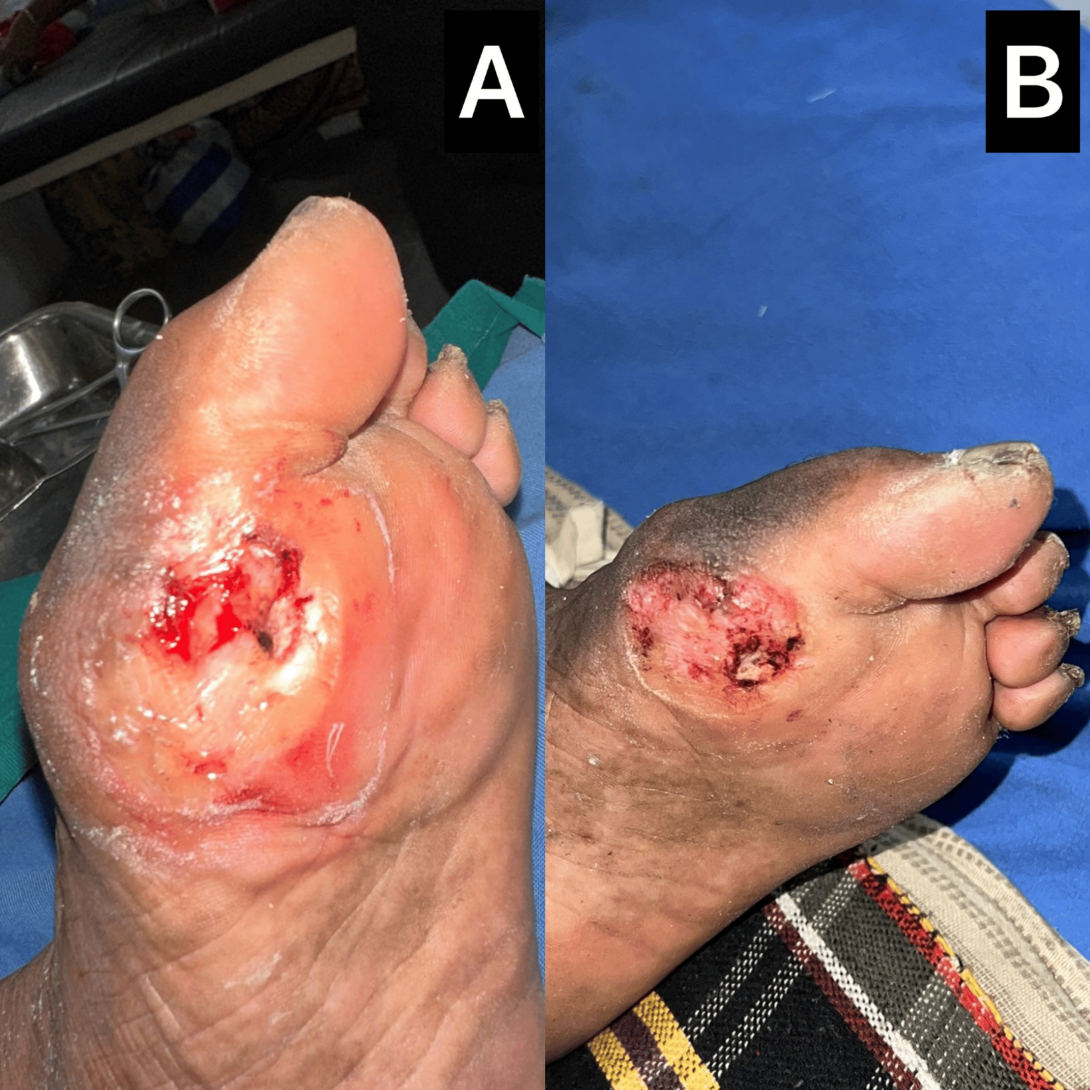
Healing of a traumatic plantar ulcer using DPA 3A: Traumatic plantar ulcer over the right great toe at presentation with slough and local inflammation; 3B: Nearly healed ulcer after three weeks of DPA application and conservative wound care DPA: diperoxochloric acid

A 64-year-old female presented with a painful ulcer on the plantar aspect of the right great toe, which developed after stepping on a sharp object while walking barefoot indoors. The wound had been present for five days and was associated with localized swelling and discomfort. Examination revealed a 2 × 2 cm shallow ulcer with slough, erythematous margins, and mild serous discharge. Peripheral pulses were palpable. Laboratory values were within normal limits. Bedside debridement was performed under local anesthesia, and the patient was started on intravenous antibiotics based on wound culture, which grew *Staphylococcus epidermidis *and *Escherichia coli*. The presence of a polymicrobial infection underscores the utility of DPA, whose broad-spectrum activity effectively addresses both gram-positive and gram-negative organisms. DPA dressings were applied daily. Over the next 10 days, the ulcer showed progressive granulation and reduction in discharge (Figure 3). No further debridement was required. The relatively rapid healing was likely facilitated by the smaller wound size and early initiation of combined topical and systemic therapy. By the end of the third week, the ulcer had almost completely healed with intact epithelialization.

Case 4

**Figure 4:**
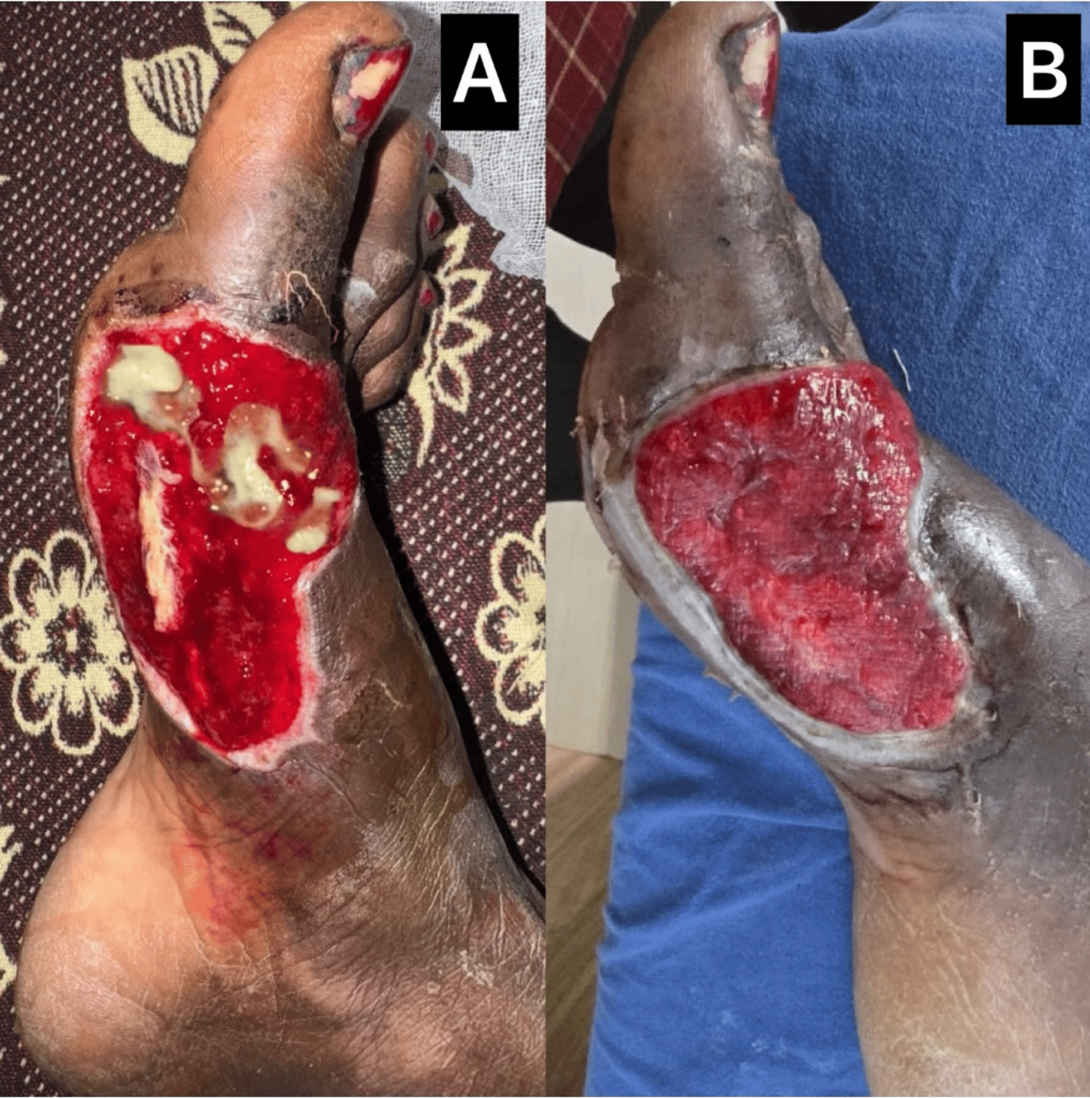
Extensive medial foot ulcer managed conservatively with DPA 4A: Infected soft tissue defect with slough and a purulent base on the dorsomedial foot at delayed presentation; 4B: Healed wound with epithelialization and wound contraction following serial debridements and topical DPA therapy over six weeks DPA: diperoxochloric acid

A 58-year-old female agricultural worker presented with a large infected ulcer over the dorsomedial aspect of her left foot, sustained 10 days earlier due to a sickle injury. Initial treatment with traditional herbal remedies led to worsening of the wound. On examination, the ulcer measured 8 × 5 cm, with a slough-covered base, purulent discharge, and exposed soft tissue layers as shown in Figure 4. Peripheral pulses were intact, and there was no evidence of osteomyelitis on X-ray. Blood investigations showed mild leukocytosis and borderline hyperglycemia. Capillary blood glucose monitoring was initiated, and dietary modifications were advised; pharmacologic intervention was not required. Surgical debridement was performed under spinal anesthesia, followed by twice-daily DPA-based wound dressings. Culture revealed *Pseudomonas aeruginosa* sensitive to ceftazidime, which was added to her regimen. Over five weeks, the ulcer showed steady granulation, reduction in size, and wound contraction. Two additional debridements were performed during this period. By the sixth week, the ulcer had epithelialized almost completely without the need for grafting or flap cover. The patient was discharged with offloading advice and wound care education.

## Discussion

In this series, all four patients exhibited wound bed preparation and closure within three to six weeks following the introduction of topical DPA as an adjunct to standard debridement and antibiotic therapy. DPA’s strong oxidizing action disrupts bacterial biofilms and inactivates microbial enzymes, aligning with the TIME framework principles of infection control and tissue preservation [1,2]. Although the TIME framework was originally developed for chronic wounds, its emphasis on debridement, infection control, and wound edge advancement translates well to traumatic ulcers, where delayed presentation and biofilm formation are common challenges [2,3]. This approach is consistent with International Working Group on the Diabetic Foot (IWGDF) guidelines, which support structured wound care across both diabetic and non-diabetic populations [3]. As summarized in Table 1, prior studies support the broad-spectrum efficacy and favorable safety profile of peracid-based antiseptics like DPA.

**Table 1:** Key literature supporting traumatic foot ulcer management

Reference	Topic	Key Findings
[1] Bal et al., 2024	DPA as novel antiseptic	Demonstrated safety and efficacy in neuropathic ulcers
[2] Leaper et al., 2012	TIME framework evolution	Emphasizes infection control, debridement, and moisture balance
[3] Lipsky et al., 2016	IWGDF guideline applicability	Management principles extend to diabetic and non-diabetic foot ulcers
[4] Jones & Edwards-Jones, 2009	Antiseptic cytocompatibility	Peracid agents cause less tissue damage than iodine or hydrogen peroxide
[5] Percival et al., 2014	pH influence on healing	Acidic pH inhibits biofilms and enhances antimicrobial activity
[6] Vowden & Vowden, 2017	Hydrogen peroxide cytotoxicity	Impairs fibroblast function at higher concentrations
[7] Guo & DiPietro, 2010	Dressing principles	Promote moist environment and epithelial advancement
[8] Serra et al., 2015	Role of P. aeruginosa & S. aureus	Major contributors to chronic wound infections
[9] Singer et al., 2016	Sepsis definitions	Consensus criteria for systemic infection detection
[10] Lavery et al., 2006	Foot infection risk factors	Include trauma, delayed presentation, and diabetes
[11] Shanmugam et al., 2013	Bacteriology in ulcers	Includes drug-resistant strains in diabetic foot ulcers
[12] Jeandrot et al., 2008	Infection biomarkers	CRP and procalcitonin help differentiate infection severity
[13] Sotto et al., 2007	Molecular diagnostics	Oligonucleotide arrays differentiate colonization vs. infection
[14] Grayson et al., 1995	Probing to bone	Indicates underlying osteomyelitis in diabetic foot ulcers
[15] Senneville et al., 2009	Bone biopsy inconsistency	Needle and transcutaneous biopsy results may be discordant

Comparative studies show that peracid compounds like DPA achieve microbial kill with minimal cytotoxicity [4], unlike agents such as povidone-iodine and hydrogen peroxide, which can impair fibroblast function at higher concentrations [6]. Wound pH plays a key role; mildly acidic environments both suppress biofilms and enhance antiseptic efficacy [5]. In our cases, moist DPA-soaked gauze dressings were used consistently, which supported the TIME principles of moisture balance and edge advancement by maintaining an optimal healing environment [7]. In our series, DPA was applied in a context likely to maintain a mildly acidic wound environment, which may have contributed to its enhanced antimicrobial efficacy.

Chronic wound infections are often caused by *Pseudomonas aeruginosa* and *Staphylococcus aureus*, which thrive in biofilms and necrotic tissue [8]. In our cases, daily DPA application following saline irrigation and dressing changes led to culture sterility within two weeks. No systemic infections occurred; sepsis was excluded based on consensus definitions [9]. Culture sterility was typically observed within two weeks and coincided with the onset of visible granulation tissue and reduction in wound size. This is particularly relevant in the polymicrobial infections seen in two of our cases, where DPA effectively addressed both gram-positive and gram-negative organisms.

Established infection risk factors include trauma, delayed care, and comorbidities like diabetes, even though our cohort had no diabetic patients [10,11]. Biomarkers such as procalcitonin and CRP help assess infection severity but were not elevated here [12]. The absence of elevated inflammatory biomarkers likely reflects the localized nature of these soft-tissue infections [13]. The absence of elevated inflammatory biomarkers likely reflects the localized nature of these soft-tissue infections.

Probing to bone and biopsy cultures are vital in suspected osteomyelitis, but have sampling limitations. Our patients showed no clinical or radiologic signs of bone involvement, so invasive tests were not pursued [14,15]. The median healing time across cases was five weeks, with a range of three to six weeks [2]. These findings align with prior evidence of DPA’s safety, ease of use, and antimicrobial efficacy [1,7], warranting larger controlled trials for protocol standardization. The main limitation of this study is the small sample size and the absence of a control group, which limits generalizability. Larger controlled trials are warranted to establish standardized protocols, including optimal DPA concentration, frequency of application, and cost-effectiveness compared to other antiseptics.

## Conclusions

This case series demonstrates that topical diperoxochloric acid (DPA), when used in conjunction with serial debridement and appropriate systemic antibiotics, can effectively control infection and accelerate healing in infected traumatic foot ulcers. Across four diverse presentations, DPA facilitated rapid granulation tissue formation, reduced wound exudate, and promoted complete epithelialization without any observed adverse events, highlighting its safety and tolerability. Incorporating DPA into the TIME framework offers a novel, practical adjunct in the management of difficult wounds. Given its ease of use and low cytotoxicity, DPA may be especially valuable in low-resource settings where access to advanced wound care products is limited. Future randomized studies are recommended to compare DPA against established antiseptics and to define its optimal concentration, application frequency, and cost‑effectiveness in varied clinical settings.
